# Mapping Retrotransposon LINE-1 Sequences into Two Cebidae Species and *Homo sapiens* Genomes and a Short Review on Primates

**DOI:** 10.3390/genes13101742

**Published:** 2022-09-27

**Authors:** Vanessa Milioto, Polina L. Perelman, Laura La Paglia, Larisa Biltueva, Melody Roelke, Francesca Dumas

**Affiliations:** 1 Department of Biological, Chemical and Pharmaceutical Sciences and Technologies (STEBICEF), University of Palermo, 90100 Palermo, Italy; 2 Institute of Molecular and Cellular Biology, Russian Academy of Sciences, 630090 Novosibirsk, Russia; 3 Bioinformatics and Computational Biology for Precision Medicine-ICAR/CNR, 90100 Palermo, Italy; 4 Leidos Biomedical Research, Inc., Frederick Bethesda, MD 20817, USA

**Keywords:** *Sapajus apella*, *Cebus capucinus*, *Homo sapiens*, LINE, rearrangements, centromere

## Abstract

This work focuses on the distribution of LINE-1 (a Long Interspersed Nuclear Element) in primates and its role during evolution and as a constituent of the architecture of primate genomes. To pinpoint the LINE-1 repeat distribution and its role among primates, LINE-1 probes were mapped onto chromosomes of *Homo sapiens* (Hominidae, Catarrhini), *Sapajus apella*, and *Cebus capucinus* (Cebidae, Platyrrhini) using fluorescence in situ hybridisation (FISH). The choice of platyrrhine species are due to the fact they are taxa characterised by a high level of rearrangements; for this reason, they could be a useful model for the study of LINE-1 and chromosome evolution. LINE-1 accumulation was found in the two Cebidae at the centromere of almost all acrocentric chromosomes 16–22 and on some bi-armed chromosomes. LINE-1 pattern was similar in the two species but only for chromosomes 6, 8, 10, and 18, due to intrachromosomal rearrangements in agreement with what was previously hypothesised as through g banding. LINE-1 interstitial accumulation was found in humans on the 1, 8, 9, 13–15, and X chromosomes; on chromosomes 8, 9, and 13–15, the signal was also at the centromeric position. This is in agreement with recent and complete molecular sequence analysis of human chromosomes 8 and some acrocentric ones. Thus, the hypothesis regarding a link between LINE-1 and centromeres as well as a link with rearrangements are discussed. Indeed, data analysis leads us to support a link between LINE-1 and inter- and intrachromosomal rearrangements, as well as a link between LINE-1 and structural functions at centromeres in primates.

## 1. Introduction

The human genome is composed of 1–2% coding regions, while 98–99% are noncoding regions; the latter are composed of variable sequences which are highly repetitive and not easily understandable, also termed the ‘dark matter’ of the genome. Mobile elements, also known as transposable elements (TEs), are recognised among the variety of repetitive elements in genomes. These sequences are quite abundant in the complex genomes of animals such as primates, and the first account has estimated they make up about 26% of the human genome [1], though more recent estimates claim they account for 45–52.1% [2,3]. With improvements in genome assemblies, it has been shown that this region is responsible for the different genome sizes, especially due to TE activity [4]. In particular, genome size variation among primates due to the presence of TEs is especially evident in Cercopithecoidea and Hominoidea, followed by Cebidae, Hylobatidae, and Lemuridae [2].

Based on the mechanism of their mobility, these transposable elements can be divided into two main classes; elements of Class I jump from one site to another through an RNA intermediate, while elements of Class II move directly without intermediaries; class I, or retrotransposons, mobilise in genomes via a “copy-and-paste” mechanism directed by reverse transcription of an RNA intermediate. This class is typically subdivided into Long Terminal Repeat (LTR) and non-LTR retrotransposons [1,3]. In particular, among non-LTR transposable elements, the Long Interspersed Nuclear Elements (LINEs) are the most abundant in primate and mammalian genomes [4,5,6]. In primate genomes, LINE abundance has been estimated to be between 16.3% and 22.5% [4].

The majority of LINEs are truncated or defective copies that were originally produced by a relatively small number of full-length, retrotransposition-competent copies [7]. Within LINEs, the elements of the family LINE-1 are the only ones which remain abundant and active in mammalian genomes, including primates [4,5], with sequence abundance representing 17–20% of the human genome [1,3,8]. The length of a LINE-1 element is about 6 kb, and it encodes an RNA-binding protein as well as a second protein with endonuclease and reverse transcriptase activity. Because they can make copies of themselves, they are likely the source of all LINE-1 elements in the genome. The human genome contains 80–100 of these retrotransposition-competent elements [7,9].

LINE-1 insertions are polymorphic (presence/absence) in the primate genome; their absence is considered the ancestral condition, and when they are present, they are identical by descent since the probability of convergence is very low [3,10,11]. Furthermore, LINE-1 is homoplasy-free in related taxa because excisions of LINE-1 are believed to be extremely rare [10,11]. For all these reasons, they can be used in population genetics, systematics, and phylogeny. For example, in this context, LINE-1 elements were studied in primates by whole-genome screening and used to infer a close phylogenetic link between *Callimico goeldii* and Platyrrhini primates [12] and to elucidate human evolutionary history [11].

Despite being considered “junk DNA” at the beginning, researchers have obtained evidence that LINE-1 elements make significant contributions to genome evolution; they are involved in DNA stability, maintaining genome integrity including DNA packaging, centromere stability, or plasticity [13,14,15,16]; furthermore, they are responsible for genome reshuffling, and they are even involved in evolution by promoting the occurrence of chromosomal rearrangements [17,18,19,20,21,22]. In addition, they can also be responsible for pathologies [23] such as haemophilia due to the insertions of LINE-1 into the factor VIII gene, resulting in target site duplications of portions of the gene and rendering it nonfunctional [24].

Despite the demonstrated importance of TE sequences, the reason for the lack of large-scale comparative studies for LINE-1 transposition in primates is due to the high content of LINEs in the primate genomes; the reference genome sequences are still incomplete or there are assembly errors, especially for the nonhuman primate genomes, due to the complex nature of the repetition of these sequences [25,26]. With improvements in genome sequencing methods and genome assemblies, TE regions will be better analysed, especially regions with high repeats such as the centromere and telomere regions, which may be hot spots for certain types of LTRs [1].

LINE-1s have been mapped in many mammals [27,28], including in primates; previous works have shown that LINE-1 is localised at centromeric and noncentromeric positions, with different patterns in the main groups of primates [15,22]. To extend LINE-1 distribution analysis to more primate samples, we used FISH to map LINE-1 probes in two platyrrhine species and in humans. The platyrrhine species are taxa characterised by a high level of rearrangements [29,30,31,32]; for this reason, they could be a useful model for the study of LINE-1 and chromosome evolution. The LINE-1 mapping results on *H. sapiens* (Hominidae, Catarrhini), *S. apella*, and *C. capucinus* (Cebidae, Platyrrhini) (Linnaeus, 1758) permit us to discuss LINE-1 evolution in a comparative perspective in light of previously published cytogenomic data. Furthermore, the sequential DAPI/CMA3 staining, FISH of LINE-1, and post-FISH C banding of chromosomes permits the evaluation of the possible correlation between heterochromatin and LINE-1 preferential insertion sites and, in a phylogenetic framework, allowed us to show many interspersed and centromeric LINE-1 signals and to hypothesis their possible role and function. 

## 2. Materials and Methods

Metaphases were obtained from fibroblast cell cultures from a male sample of *S. apella* and *C. capucinus* (Cebidae), from Catoctin Zoo, Thurmont MD, USA and the Laboratory of Genomic Diversity of the National Cancer Institute, Frederick, MD, USA. Fibroblast cells were grown for 72 h in alphaMEM culture medium (Gibco, Waltham, MA, USA), 5% Antibiotics Penicillin/Streptomicin, 15% FBS, 10% amniomax (Gibco).

Lymphoblasts of a male sample of *H. sapiens* were grown in RPMI culture medium, following standardised protocols to obtain metaphases. 

Cells harvesting was performed after 3 h incubation of colcemid 10 μL (10 μg/mL Gibco) followed by hypotonic treatments 0.075 M KCl for 20 min at 37 °C following a protocol from Small et al. [33].

### 2.1. Karyotyping and Sequential Chromosome Staining

Metaphases of the analysed species were stained pre- and post-FISH using chromomycin A3 -CMA3 and 4′,6-diamidin-2-fenilindolo -DAPI staining, according to a recent protocol [34], with some adjustments. CMA3 staining of GC-rich regions and DAPI staining of AT-rich regions were useful for identifying chromosomes and preferential insertion sites of LINE-1 sequences. DAPI images were inverted with a photo editing program (Adobe Photoshop C 2022 V23.3.2); inverted grey bands generally correspond to dark G-bands or light R bands; the DAPI inverted karyotypes for the *S. apella* and *C. capucinus* species were compared with previously published R- or G-banded karyotypes [35,36,37]. It is necessary to emphasise that *S. apella* was previously recognised as *Cebus apella* (see [38] for a review).

C-banding was performed sequentially post-FISH through a protocol that included denaturation with formamide [39], and the C-banded karyotypes were compared with previously published data [37].

### 2.2. LINE-1 Probe Preparation

DNA extraction from the fibroblast cell lines was performed using the Pure Link DNA kit (Invitrogen, Waltham, MA, USA) according to the basic DNA extraction protocol. LINE-1-like repeat sequences called LINE-1 have been amplified by polymerase chain reaction (PCR); each probe was amplified from the species own DNA; the universal set of primers, developed for the PCR of LINE-1 repeats in mammals, have been used: L1R, 5′-ATTCTRTTC CAT TGG TCT A-3′ and L1F 5′-CCA TGC TCATSGAT TGG -3′ [40,41].

Genomic DNA was amplified in 50 μL PCR-reactions: five units of Taq GOLD DNA Polymerase (Invitrogen), the template DNA, 500 nM of each primer, 200 μM each of dATP, dCTP, dTTP, and dGTP in 10 mM TRIS-HCl, pH 8.3, 1.5 mM MgCl_2_, 50 mM KCl. PCR reactions were performed using an Applied biosystems SimplyAmp (Thermo Fisher Scientific, Waltham, MA, USA) with the following cycling parameters: 30 cycles each of 94 °C, 30 s; 52.5 °C, 30 s; 72 °C, 30 s, following a 2 min denaturation at 94 °C. A bright band of about 400 pb was visualised on 1% agarose gel. The PCR products were directly labelled through nick translation using 11-dUTP-Fluorescein (green) (Invitrogen) for *H. sapiens* and dUTP-cy5 (red) (Amersham) for the Cebidae species.

### 2.3. Fluorescent In Situ Hybridisation (FISH)

FISH was performed following previously described protocols using LINE-1 probes obtained by PCR [42,43]. The hybridisation mix consisted of 2.5 ng/L of probe, 50% formamide, 10% dextran sulphate, and 2XSSC, with an incubation time of 18 h at 37 C. Detection was performed at high stringency with washing at high temperatures (68 °C) and at low saline buffer concentration of 0.4 and 2XSSC.

### 2.4. Genomic Browser

The genomic browser UCSC has been used to extrapolate data on LINE-1 localisation in *H. sapiens*. Furthermore, using the hub_2004795_RepeatMasker available through USCS, we downloaded annotation tracks of LINE-1-related repeated elements for the analysed species.

## 3. Results

### 3.1. LINE-1 Distribution of Chromosomes of Cebidae and H. sapiens

After FISH, the metaphases were analysed under a Zeiss Axio2 epifluorescence microscope. Images were captured using a coupled Zeiss digital camera. At least ten methaphase spreads were analysed for each sample. Chromosomes were classified according to the proposed nomenclature [44].

**Figure 1:**
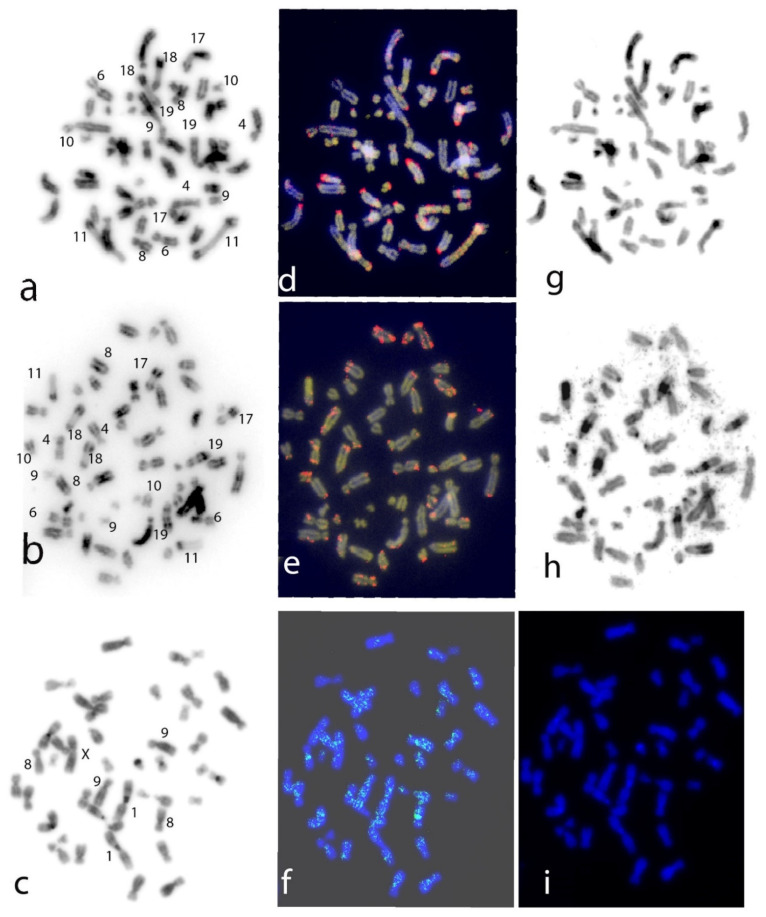
Examples of sequential stains and LINE-1 FISH mapping in the analysed species; from left: DAPI inverted staining before FISH (some representative chromosome pairs have been identified with numbers, see Figure 2 for the complete karyotypes), DAPI/CMA3 (blue/yellow) stains/LINE-1 probe localisation pattern (red) in overlapping, and the C banding after FISH onto the same mitotic metaphase, respectively, in *S. apella* (SAP), (**a**,**d**,**g**) and in *C. capucinus* (CCP), (**b**,**e**,**h**). In *H. sapiens* (HSA); from left: C banding after FISH (**c**), LINE-1 probe localisation pattern (green) (**f**), DAPI staining after FISH (**i**).

LINE-1 probe mapping revealed bright signals on the metaphases of the three species analysed, with a different accumulation pattern (Figure 1d,e,f). Sequential staining, banding, and FISH mapping was performed for the two Cebidae species (Figure 1a,d,g in SAP; Figure 1b,e,h in SAP).

**Figure 2:**
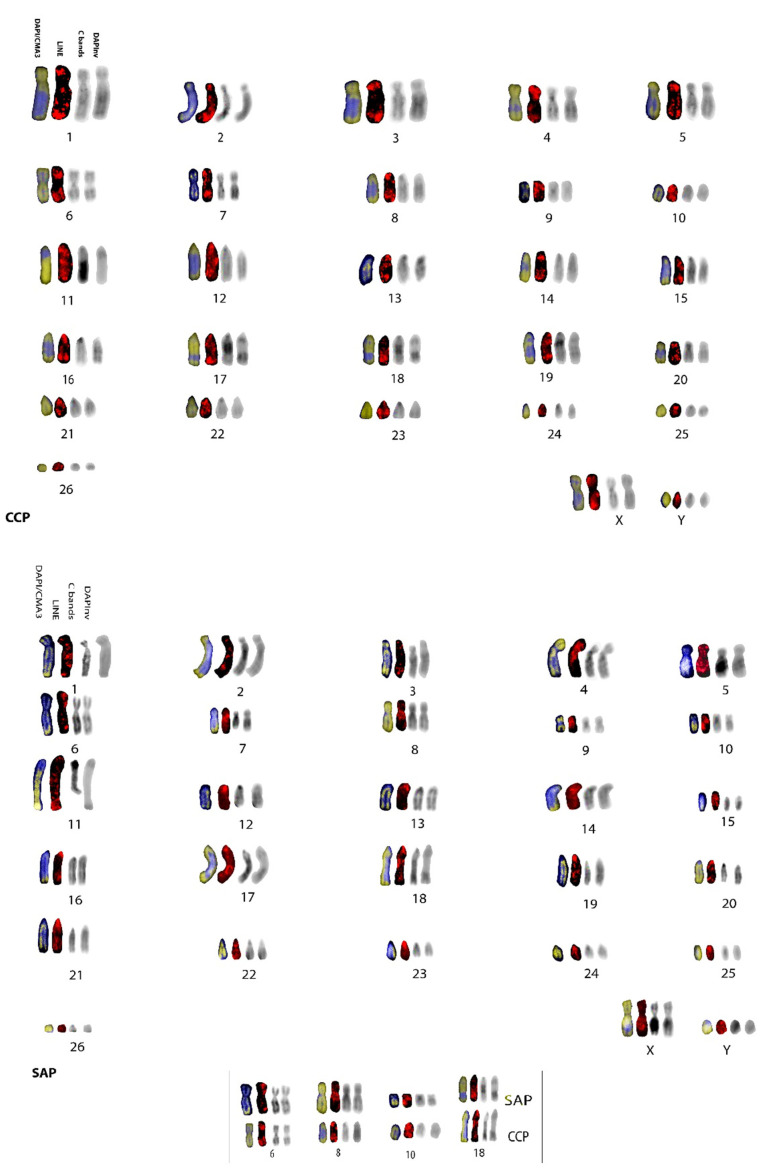
Haploid karyotypes of *C. capucinus* (CCP) and *S. apella* (SAP), from left: chromosomes with DAPI staining (blue) and CMA3 (yellow) overlapped; LINE probe signals (red); C bands; inverted DAPI (black and white). Note in the box chromosomes 6, 8, and 10 differently due to inversions in SAP and CCP; additionally, pair 18 has different patterns of both C bands and LINE-1 signals.

Karyotypes were reconstructed accordingly (Figure 2), using the same metaphases shown in Figure 1. The inverted DAPI karyotypes of *C. capucinus* and *S. apella* were in agreement with previously published ones [35,36,37], with both species having the diploid number 2n = 54; we followed a previous reconstruction [37] for chromosome numbering. The two Cebidae species showed almost the same karyotype (Figure 2), with ten pairs of meta/submetacentric chromosomes in *S. apella* (pairs 1–10), eight pairs in *C. capucinus* (1–7, 9), and fourteen and sixteen acrocentric chromosomes, respectively, thus differing over chromosome pairs 8 and 10 that are submetacentric in *S. apella* and acrocentric in *C. capucinus*.

DAPI/CMA3 staining was helpful for identifying chromosomes and preferential sites of LINE-1 insertion (Figure 1d,e and Figure 2). The post-FISH C- banding pattern of the two Cebidae species (Figure 1g,h and Figure 2) was in agreement with previously published C-banding patterns obtained by classic C-banding [37]. We showed that C bands are at the centromeres of almost all chromosome pairs and at the peculiar interstitial bands on chromosomes 11 and 17–19 (Figure 1g,h and Figure 2).

**Figure 3:**
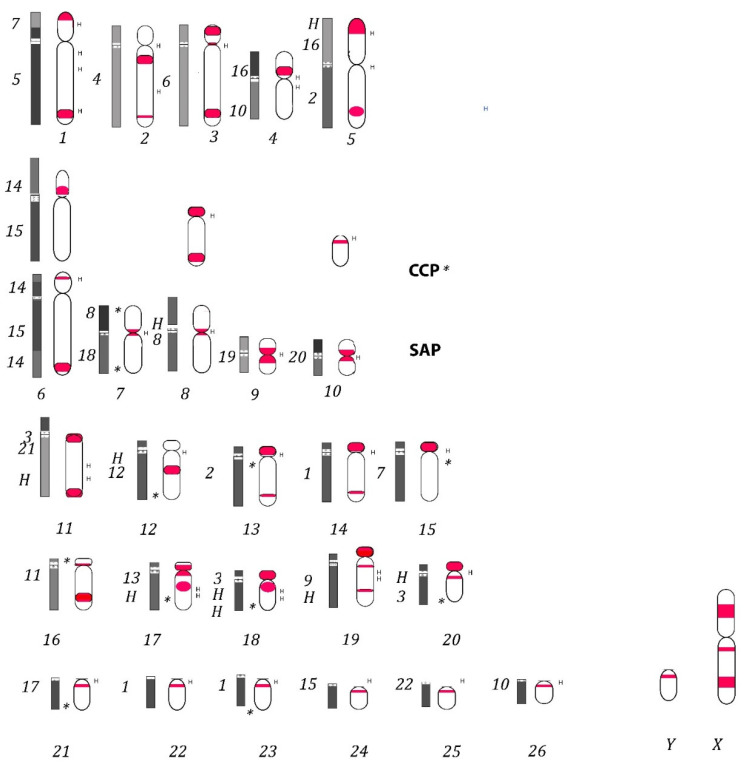
Haploid ideograms for the two species: *S. apella* and *C. capucinus* (SAP and CCP), with LINE-1 signals in red. Note chromosome pairs 6, 8, and 10 are different due to inversions between the species (CCP chromosomes 6, 8, and 10 forms are reported above the line); additional signals are indicated with an asterisk for CCP *. The human syntenies as well as constitutional heterochromatin–H extrapolated from previous painting data are reported at the left of chromosomes with numbers and bars [35,36,37]; different human associations on the same chromosomes are reported on bars also with grey or black colors. At the right of each ideogram the H indicates C- bands here obtained. Chromosomes 17 and 18, apart from a different LINE-1 signal pattern, have a slightly different C banding, not reported here (see also Figure 2).

In the two Cebidae species, LINE-1 signals can be observed at the centromeres of some submetacentric chromosomes; for example, on chromosomes 8 and 10 in *S. paella* (Figure 1 and Figure 2), chromosome 9 in both species and especially on acrocentric chromosomes (13–26) in both species, but also interstitially along chromosome arms and at the terminal ends of chromosomes (Figure 2 and Figure 3). The X chromosome is evenly rich in LINE-1 along both arms. LINE-1 on the Y chromosome shows a pericentromeric distribution (Figure 2 and Figure 3).

Homologies with human syntenies were taken into account by extrapolating data from painting results for karyotypes of *C. capucinus* and *S. apella* [35,36,37] (Figure 3).

LINE-1 probe signals on human metaphases (Figure 1c) were depicted on the human DAPI inverted karyotype (Supplementary Figure S1). LINE-1 probes showed bright signals especially prominent at the centromere of chromosome pair 8 and along chromosome 1 and X, and slight signals showed on chromosome pairs 9 and 13–15. Signals have been reported analysing more metaphases because some signals are not always evident.

### 3.2. Genomic Browser Data

The data on repetitive sequences including LINE-1 for the analysed species extrapolate from the UCSC genome browser (Supplementary Files S1, S2, and S3) have been discussed in a cytogenomic contest and in part depicted on Supplementary Figure S1. 

## 4. Discussion

The current consensus view of primate phylogeny divides the primate order into two suborders: Strepsirrhini and Haplorhini. Strepsirrhini includes the Lorisiformes (lorises) and Lemuriformes (lemurs). Haplorrhini is further subdivided into the Platyrrhini (New World monkeys, NWMs), Catarrhini, composed of Cercopithecidae (Old World monkeys, OWMs), and Hominoidea (apes and humans) [45]. Here, two NWMs and an OWM were analysed by FISH, while a short cytogenomic review is reported for the primates analysed so far.

### 4.1. FISH Data Analysis of LINE-1 in the Analysed Species

Among primates, Platyrrhini are NWMs characterised by a high level of chromosome rearrangements [38] and thus represent a good model for studying whether LINE-1 sequences could be linked to genome evolution. Here, previous investigations [15,22] through the FISH mapping of the LINE-1 probe onto chromosomes of two NWMs, *S. apella* and *C. capucinus* (Cebidae), and of *H. sapiens* (Hominoidea), were expanded to better analyse LINE-1′s distribution and role among primate species’ genomes. The LINE-1 pattern was compared with those previously published for NWMs [15,22] to investigate possible evolutionary implications and preferential insertion sites. LINE-1 and C patterns obtained for the two NWMs are reported on ideograms in Figure 3; in the two *Cebidae* species analysed in the present work, we found an accumulation of LINE-1 elements displaying a nonrandom distribution by accumulating primarily in CMA-3 and C- positive bands at centromeres or pericentromeric regions (chromosome pairs 13–26 in both species, with some exceptions) (Figure 1a–h, Figure 2 and Figure 3). This result is in agreement with what was previously shown in many other mammals, such as bats, rodents [27,28], and primates [15,22,40,41,46,47,48]. The comparison of the LINE-1 mapping with previously published data, in particular in species from the Cebidae family, such as *Saguinus midas, S. bicolour* [41], S. *mystax, Leontocebus fuscicollis, Leontopithecus rosalia* [15], *Aotus nancymaae,* and an Atelidae, *Alouatta belzebul* [22]*,* showed predominantly centromeric distribution in all species. LINE-1 localisation at centromeres or pericentromeric regions in CMA3 and C positive bands seems to be an ancestral situation present on almost all platyrrhini chromosomes studied so far, indicating that this accumulation may have occurred in the common ancestor of all Platyrrhini, contributing to the current features of their karyotype. These signals at centromeres possibly indicate that LINE-1 can have a preferential site of integration in these locations in Platyrrhini where the classic α satellite DNA [46] are present.

Apart from signals at centromeres, noncentromeric LINE-1 signals were found along chromosomal arms on the X chromosome and on autosomes in the two analysed NWM species (Figure 1a–h, Figure 2 and Figure 3) in euchromatic regions, both in DAPI and in CMA3-positive regions, in agreement with what was observed in a few mammalian groups [20,27,28,40], including the previously analysed platyrrhini species [22].

From an evolutionary perspective, LINE-1 signals found along chromosomes at noncentromeric regions, through a comparison with the supposed human chromosomal homologies reported for *C. capucinus* and *S. apella* [37] (Figure 3), led us to hypothesise that these repetitive elements are presumably linked to rearrangements, which is in agreement with what was already observed in other New World primates [19,20,22]; LINE-1 is located in breakpoint regions at the junction of human syntenic blocks and may be linked to ancestral–recent fusion events or to intrachromosomal rearrangements. For example, bright LINE-1 signals were found on chromosomes 4 and 6 which are covered, respectively, by human ancestral platyrrhini and primate syntenies, respectively, 16/10 and 15/14 (Figure 2 and Figure 3) [38]. Moreover, most of the interstitial LINE-1 signals also have just a partial colocalisation with C bands in the analysed species (Figure 2 and Figure 3), which is in agreement with results obtained from other platyrrhini species [22].

Furthermore, other human chromosome homologues in these platyrrhini species are subject to intrachromosomal rearrangements, and the LINE-1 signals reflect rearrangements, for example, on chromosome pairs 6, 8, and 10 (Figure 2 and Figure 3); chromosome 6 shows different DAPI inverted and CMA3 as well as LINE-1 hybridisation in the two Cebidae; the chromosome is almost submetacentric in *S. apella* and subtelocentric in *C. capucinus* as a consequence of a pericentric inversion, which is in agreement with previously reported G-banding analysis [37]. Furthermore, these results are in agreement with previous painting data through which chromosome 6 was shown to be covered by the 14/15/14 syntenic association for *S. apella*, but only 15/14 for *C. capucinus*, presumably as a result of the intrachromosomal rearrangements; LINE-1 signals were indeed located, respectively, on the distal position of the p and q arms in the former and at pericentromeric positions with a less-defined amplification in the latter (Figure 2 and Figure 3). In addition, due to a pericentric inversion, chromosome pair 8 has a different morphology, being acrocentric in *C. capucinus* and submetacentric in *S. apella*, which is in agreement with previous data [37], and the LINE-1 pattern reflects this difference. Indeed, the LINE-1 signal is at both terminal positions of the acrocentric chromosome or at the centromere of the submetacentric form. Chromosome pair 10 has a different morphology and LINE-1 signal pattern too, being acrocentric in *C. capucinus* and submetacentric in *S. apella* as a consequence of another inversion, as previously hypothesised [37]. Indeed, the LINE-1 signal is at both the terminal ends of the acrocentric form, while it is at the centromeric position of the submetacentric form (Figure 2 and Figure 3).

Other LINE-1 signals have also been shown in both species in regions rich in interspersed heterochromatin; for example, on chromosome pairs 17 and 18, slightly different pattern of C bands have been shown between the two species, with the LINE-1 probe-mapping pattern reflecting these differences (Figure 2 and Figure 3). Indeed, in *C. capucinus* LINE-1 signal is interstitial while it is at terminal position distal to centromere in *S. paella.*

Among primates, Catarrhini are OWMs, including *H. sapiens* (HSA). The first attempts to use FISH to map a LINE-1 probe on the human genome was for an incomplete sequence; this early work reported signals with a banding pattern on AT regions [49], but comparison with our LINE-1 mapping is not applicable. In the present work, we mapped the complete LINE-1 sequence on human metaphases and, through inverted DAPI, identified the chromosomes where the probe showed brightly defined signals. We found bright signals in euchromatic and heterochromatic regions, especially on chromosome pair 8, both at the centromeric CG rich region and also interspersed along chromosomes. Other interspersed signals were observed on the X and other chromosomes, for example, on the submetacentric chromosome pairs 1 and 9, and on acrocentric chromosome pairs 13–15, but in the former chromosomes at a lower level of accumulation not clearly detectable at the cytogenetic level of resolution (Figure 1f and Supplementary Figure S1). Moreover, the higher accumulation of LINE-1 signals on the X chromosome compared with autosomes is in agreement with previous FISH LINE-1 mapping in *H. sapiens* and other mammals [40].

### 4.2. Cytogenomic Data Analysis of LINE-1 in Primates

In this work, apart from mapping LINE-1 probe distribution using FISH on representative species of anthropoid primates, molecular cytogenomic data including sequence and FISH analysis of LINE-1 in primate genomes were reviewed. 

Table 1 summarises the species (and corresponding references) where LINE-1 sequences have been mapped

LINE-1 originated well before the origin of primates (at least 170 mya). Three classes of LINEs have been recognised at about 70 mya; however, already at about 40 mya in Anthropoid primates, only one class remains—LINE-1 [11,50,51]. In primates, apart from LINE-1 insertions predating the origin of primates, there are more recent primate-specific insertions. Accordingly, the retrotransposon composition of primate genomes is represented by both old elements and new ones. Due to their transposition replication, insertion elements can be truncated and classified into families based on the shared nucleotide differences they inherit from their common ancestor [52].

The LINE-1 elements amplified during primate radiation [7,53,54] are linked with the physical expansion of primate genomes. Variations in copy number of these LINE-1 elements are responsible for the larger genome size in anthropoid primates compared to prosimian primates [11,12,55,56]. Among Catarrhini, LINE-1 sequence comparison in anthropoids, including humans, demonstrated a high rate of LINE-1 amplification; in hominoidea, five subfamilies of LINE-1 arose, starting from 25 mya [51,52,57]. The rhesus macaque genome could be considered an exception, as no retrotransposition-competent LINE-1 elements have been recognised [58]. Recently, over the last 6 million years, mobilised LINE-1 has been detected with different rates in chimpanzees and humans [3,11,50,51,53,54,59]. Most transposition-competent human LINE-1 elements belong to a subset called Ta. Ta elements first appeared ∼4 million years ago or later [2]. Other studies suggest that rates of LINE-1 amplification differ substantially between the *Homo* and *Pan* lineages, indicating that LINE-1 amplification may have changed rapidly during primate evolution [5]. Full-length LINE-1 sequences have also been detected in Platyrrhini [5,60]. In particular, high LINE-1 activity has also been shown in the *Saimiri* and *Saguinus* genera through sequence comparisons in NWMs, but results in the *Ateles* lineage are in conflict [5,60]. 

Recent comparative studies have analysed raw LINE-1 elements uniquely owned by each primate genome showing different rates of accumulation. In particular, raw numbers of LINE-1 elements have been estimated for chimpanzees (5913), orangutans (21,711), crab-eating macaques (782), rhesus macaques (3016), green monkeys (11,981), and humans, based on the most updated reference sequences [1,61]. Other detailed analyses have been recently performed on single species, for example, *Pan troglodytes* [62] and *Gorilla gorilla* [63], while human-specific elements were previously found [7] and are still being researched [1]. In humans, it has been demonstrated that LINE-1 are also responsible for the interindividual variability linked to structural variants, providing a large number of insertions that are informative for fine-scale analysis of human genetic population history [12], even in extinct hominid species [64]. 

In the present work using hub_2004795_RepeatMasker available through USCS, annotation tracks for LINE-1-related repeated elements for genome assembly of *C. capucinus*/*S. apella* GSC_monkey_1.0 Dec. 2019 (GCF_009761245.1) and *H. sapiens/*GCF_000001405.39_GRCh38.p13, Feb 2019, were analyzed. In the NWMs, a kind of L1ME3G#LINE/L1 with a count of 1,423,257 was found (Supplementary File S1). For humans, two kinds of LINE-1, L1ME3C#LINE/L1 and L1MC4a#LINE/L1, with a count of 1,606,379 were found (Supplementary File S2). (The NW_022436941.1 LINE-1 sequence is reported in Supplementary File S3). Furthermore, to analyse LINE-1 region locations in the human genome, the UCSC genome browser was here used, through which LINE-1 accumulation has been found, especially on chromosomes 1 (1p 32 and q 32.1), 8 (8q21.3), 9 (9q21.3), 13 (13q 14.2) 14, (14 q23.1), and X (Xp22. 33), which is in agreement with the FISH results (Figure 1 and Supplementary Figure S1); indeed, in the browser, LINE-1 related regions are reported on the same chromosomes showed by FISH (Supplementary Figure S1), even though the centromeric signals are missed by classic sequencing method.

However, if the classic sequencing methods do not easily permit the detection of repeated regions, including LINE-1, a current, sensitive method, CHM13h (haploid CHM13h TERT cell line, originally isolated from a hydatidiform mole) permits the study of highly repetitive genomic regions such as centromeres [65,66]; this method applied to human chromosome 8 demonstrated LINE-1 to be present at the centromere of human chromosome 8 [65] and of acrocentric chromosomes [66] (data reported in Supplementary Figure S1); in agreement with FISH mapping of LINE-1 at centromeric position in *Homo*. Furthermore, species-specific TEs have been detected in gibbon genomes at the centromere position too. There are TEs consisting of pieces of LINE-Alu- and VNTR-Alu-like, a nonautonomous composite element that can be mobilised by LINE-1, named LAVA, in the gibbon genomes [67,68]. This kind of element is not unique to gibbons as a similar TE, KERV (kangaroo endogenous retrovirus), has been detected also in the wallaby species [69].

## 5. Conclusions

The present work shows a rich content of LINE-1 for the two NWM species analysed, in agreement with previous molecular cytogenetic data in other Cebidae [15,22,38]; in humans, a lower amplification pattern has been observed. The high presence of LINE-1 in these NWMs also agrees with results of previous molecular sequence data analysis in which high LINE-1 activity was shown for other Cebidae species, including *Saimiri* and *Saguinus* [5,60].

LINE-1 distribution in the NWM species analysed can be summarised as follows: LINE-1 signals are at the junction of some human syntenic associations, which is in agreement with previous molecular cytogenetic analysis [22]; this localisation supports the hypothesis that links LINE-1 and chromosome rearrangements [20,40]. 

LINE-1 signals are at the centromeric position, as already seen in other Platyrrhini [22]; FISH has also shown LINE-1 at centromeres on some human chromosomes. This evidence is in agreement with recent CHM13h data; indeed, the presence of LINE-1 at the centromere of human chromosome 8 and other acrocentric chromosomes [65,66] has been shown; this evidence supports the hypothesis of LINE-1 being involved in the centromere structure. 

Despite all this evidence, further multidisciplinary approaches, including the comparison of sequence analysis and FISH mapping in many other species, are needed to test previous hypotheses and better define the precise role and function of LINE-1.

## Figures and Tables

**Table 1:** List of previously primate species analysed by FISH with LINE-1 probes.

	Latin Name/Code	Reference
Platyrrhini		
	*A. nancymaae*, ANA *A. belzebul*, ABE	[15]
	*L. rosalia*, LRO *Leontocebus fuscicollis*, LFU *Saguinus mystax*, SMY	[22]
	*S. apella*, SAP *C. capucinus*, CCP	[present work]
	*Saguinus midas*, SMI *Saguinus concolor*, SCO	[39,41]
Catarrhini	*H. sapiens*, HSA	present work, [40,49]

## Data Availability

Not applicable.

## Supplementary Materials

The following supporting information can be downloaded at: https://www.mdpi.com/article/10.3390/genes13101742/s1, Figure S1. Diploid karyotype of *H. sapiens*, from left: LINE-1 probe signals (green), DAPI staining (blue), inverted DAPI (black and white); File S1: Annotation tracks for LINE-1-related repeated elements for *C. capucinus/Sapajus paella* obtained using hub 2004795 RepeatMasker available through USCS; File S2: Annotation tracks for LINE-1-related repeated elements for *H. sapiens* obtained using hub 2004795_RepeatMasker available through USCS; File S3: The NW_022436941.1 LINE-1 sequence from *Sapajus paella* genome.
