# Supplementary material for: Mapping Retrotransposon LINE-1 Sequences into Two Cebidae Species and Homo sapiens Genomes and a Short Review on Primates
Source: Genes (Basel). 2022 Sep 27;13(10):1742. doi: 10.3390/genes13101742 (PMC9601419; doi:10.3390/genes13101742)
Supplement: Supplementary file 1 [file genes-13-01742-s001.zip › Supplementary File S1 UCSC GCF, sap Schema for RepeatMasker - RepeatMasker Repetitive Elements.pdf]

## Schema for RepeatMasker - RepeatMasker Repetitive Elements

**Database:** hub_2004795_GCF_009761245.1 **Primary Table:** hub_2004795_repeatMasker **Data last updated:** 2022-05-19
**Big Bed File:** https://hgdownload.soe.ucsc.edu/hubs/GCF/009/761/245/GCF_009761245.1/bbi/GCF_009761245.1_GSC_monkey_1.0.rmsk.bb
**Item Count:** 4,268,248
**Format description:** Repetitive Element Annotation

| field | example | description |
|---|---|---|
| chrom | NW_022436941.1 | Reference sequence chromosome or scaffold |
| chromStart | 60400402 | Start position of visualization on chromosome |
| chromEnd | 60403764 | End position of visualation on chromosome |
| name | L2c#LINE/L2 | Name repeat, including the type/subtype suffix |
| score | 370 | Divergence score |
| strand | + | + or - for strand |
| thickStart | 60403385 | Start position of aligned sequence on chromosome |
| thickEnd | 60403695 | End position of aligned sequence on chromosome |
| reserved | 0 | Reserved |
| blockCount | 3 | Count of sequence blocks |
| blockSizes | 2982,310,69 | A comma-separated list of the block sizes(+/-) |
| blockStarts | -1,2983,-1 | A comma-separated list of the block starts(+/-) |
| id | 83235 | A unique identifier for the joined annotations in this record |
| description | 313 37.000 11.000 2.400 NW_022436941.1 60403386 60403695 (30201586) + L2c LINE/L2 2983 3318 (69) 83235 | A comma separated list of technical annotation descriptions |

## Sample Rows

| chrom | chromStart | chromEnd | name | score | strand | thickStart | thickEnd | reserved | blockCount | blockSizes | blockStarts | id | |
|---|---|---|---|---|---|---|---|---|---|---|---|---|---|
| NW_022436941.1 | 60400402 | 60403764 | L2c#LINE/L2 | 370 | + | 60403385 | 60403695 | 0 | 3 | 2982,310,69 | -1,2983,-1 | 83235 | 313 37.000 11.000 2.400 NW_022436941.1 6040338 |
| NW_022436941.1 | 60403298 | 60404052 | Tigger13a#DNA/TcMar-Tigger | 233 | - | 60403700 | 60403980 | 0 | 3 | 402,280,72 | -1,402,-1 | 83236 | 988 23.300 6.400 0.300 NW_022436941.1 60403701 |
| NW_022436941.1 | 60403987 | 60409119 | L2c#LINE/L2 | 317 | - | 60403987 | 60405903 | 0 | 5 | 0,65,-1,144,3216 | -1,0,-1,1772,-1 | 83237 | 198 28.000 0.000 6.600 NW_022436941.1 60403988 |
| NW_022436941.1 | 60404427 | 60404845 | LTR79#LTR/ERVL | 281 | + | 60404440 | 60404812 | 0 | 5 | 12,94,175,205,33 | -1,13,-1,180,-1 | 83238 | 258 33.000 0.000 0.000 NW_022436941.1 60404441 |
| NW_022436941.1 | 60404498 | 60404578 | MADE1#DNA/TcMar-Mariner | 57 | - | 60404543 | 60404578 | 0 | 3 | 45,35,0 | -1,45,-1 | 83239 | 264 5.700 0.000 0.000 NW_022436941.1 60404544 6 |
| NW_022436941.1 | 60404563 | 60405293 | Eutr16#DNA?/hAT-Tip100? | 254 | - | 60404932 | 60405293 | 0 | 3 | 369,361,0 | -1,369,-1 | 83240 | 664 25.400 13.800 5.100 NW_022436941.1 6040493 |
| NW_022436941.1 | 60405273 | 60405482 | MIR3#SINE/MIR | 312 | + | 60405309 | 60405373 | 0 | 3 | 35,64,109 | -1,36,-1 | 83241 | 260 31.200 0.000 0.000 NW_022436941.1 60405310 |
| NW_022436941.1 | 60405461 | 60408881 | L2a#LINE/L2 | 207 | - | 60405462 | 60405549 | 0 | 3 | 1,87,3332 | -1,1,-1 | 83242 | 356 20.700 6.900 0.000 NW_022436941.1 60405463 |
| NW_022436941.1 | 60405561 | 60411717 | L1ME3G#LINE/L1 | 176 | - | 60405575 | 60405684 | 0 | 3 | 14,109,6033 | -1,14,-1 | 83243 | 226 17.600 20.200 0.800 NW_022436941.1 6040557 |
| NW_022436941.1 | 60405929 | 60406235 | AluJo#SINE/Alu | 173 | + | 60405930 | 60406219 | 0 | 3 | 0,289,16 | -1,1,-1 | 83244 | 1791 17.300 2.800 0.300 NW_022436941.1 6040593 |

## RepeatMasker (hub_2004795_repeatMasker) Track Description

# Description

This track shows the Repeat Masker annotations on the 17 Dec 2019 *Sapajus apella*/GCF_009761245.1_GSC_monkey_1.0 genome assembly.

This track was created by using Arian Smit's RepeatMasker program, which screens DNA sequences for interspersed repeats and low complexity DNA sequences. The program outputs a detailed annotation of the repeats version of the query sequence in which all the annotated repeats have been masked (generally available on the Downloads page). RepeatMasker uses the Repbase Update library of repeats from the Genetic Information I section below.

# RepeatMasker and libraries version

[illegible]

```
The repeat files provided for this assembly were generated using RepeatMasker.
  Smit, AFA, Hubley, R & Green, P.,
  RepeatMasker Open-3.0.
  1996-2010 .

VERSION:
RepeatMasker version open-4.0.8 , sensitive mode
run with blastp version 2.0MP-WashU [01-Jan-2006] [linux24-i786-ILP32F64 2006-01-02T05:13:21]
RepeatMasker Combined Database: Dfam_Consensus-20181026, RepBase-20181026

PARAMETERS:
RepeatMasker -engine wublast -species 'sapajus apella' -s -no_is -cutoff 255 -frag 20000

REPEATS:
RepeatMasker Database: RepeatMaskerLib.embl
Version: RepeatMasker Combined Database: Dfam_Consensus-20181026, RepBase-20181026
Species: sapajus apella ( sapajus apella )
1376 ancestral and ubiquitous sequence(s) with a total length of 1363805 bp
0 sapajus apella specific repeats with a total length of 0 bp
0 lineage specific sequence(s) with a total length of 0 bp
-------------------------------------------------------------------------------
```

## Display Conventions and Configuration

### Context Sensitive Zooming

This track employs a technique which chooses the appropriate visual representation for the data based on the zoom scale, and or the number of annotations currently in view. The track will automatically switch from the mo
is greater than 45kb of sequence. It will further switch to the even denser single line view ('Dense' mode) if more than 500 annotations are present in the current view.

### Dense Mode Visualization

In dense display mode, a single line is displayed denoting the coverage of repeats using a series of colored boxes. The boxes are colored based on the classification of the repeat (see below for legend).

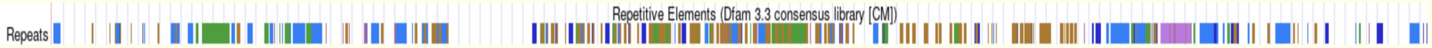


### Pack Mode Visualization

In pack mode, repeats are represented as sets of joined features. These are color coded as above based on the class of the repeat, and the further details such as orientation (denoted by chevrons) and a family label are p

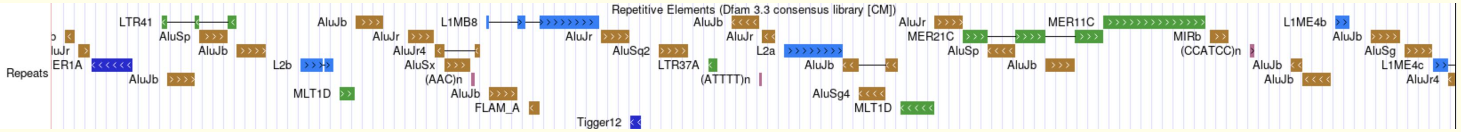


The pack display mode may also be configured to resemble the original UCSC repeat track. In this visualization repeat features are grouped by classes (see below), and displayed on seperate track lines. The repeat range
base mismatch, base deletion, and base insertion associated with a repeat element. The higher the combined number of these, the lighter the shading.

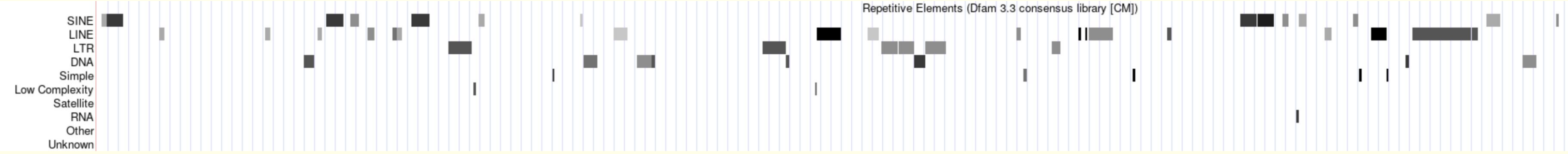


### Full Mode Visualization

In the most detailed visualization repeats are displayed as chevron boxes, indicating the size and orientation of the repeat. The interior grayscale shading represents the divergence of the repeat (see above) while the outli or right indicate the length of unaligned repeat model sequence and provide context for where a repeat fragment originates in its consensus or pHMM model. If the length of the unaligned sequence is large, an iterruption li

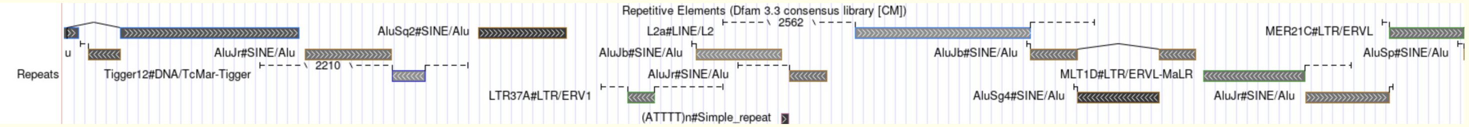


For example, the following repeat is a SINE element in the forward orientation with average divergence. Only the 5' proximal fragment of the consensus sequence is aligned to the genome. The 3' unaligned length (384bp) length of the unaligned sequence.

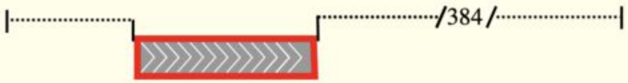


Repeats that have been fragmented by insertions or large internal deletions are now represented by join lines. In the example below, a LINE element is found as two fragments. The solid connection lines indicate that there form the 3' extremity of the repeat, as there is no unaligned consensus sequence following the last fragment.

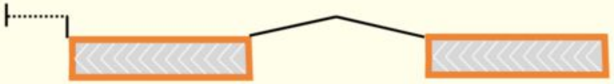

In cases where there is unaligned consensus sequence between the fragments, the repeat will look like the following. The dotted line indicates the length of the unaligned sequence between the two fragments. In this case fragments.

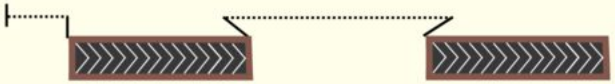

If there is consensus overlap between the two fragments, the joining lines will be drawn to indicate how much of the left fragment is repeated in the right fragment.

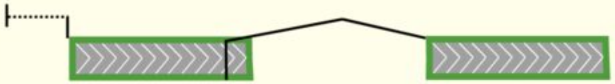

The following table lists the repeat class colors:

| Color | Repeat Class |
|---|---|
| | **SINE - Short Interspersed Nuclear Element** |
| | **LINE - Long Interspersed Nuclear Element** |
| | **LTR - Long Terminal Repeat** |
| | **DNA - DNA Transposon** |
| | **Simple - Single Nucleotide Stretches and Tandem Repeats** |
| | **Low_complexity - Low Complexity DNA** |
| | **Satellite - Satellite Repeats** |
| | **RNA - RNA Repeats (including RNA, tRNA, rRNA, snRNA, scRNA, srpRNA)** |
| | **Other - Other Repeats (including class RC - Rolling Circle)** |
| | **Unknown - Unknown Classification** |

A "?" at the end of the "Family" or "Class" (for example, DNA?) signifies that the curator was unsure of the classification. At some point in the future, either the "?" will be removed or the classification will be changed.

## Methods

## Methods

The RepeatMasker (www.repeatmasker.org) tool was used to generate the datasets found on this track hub.

## Class profiles

- 1,698,129 - SINE
- 1,423,257 - LINE
- 668,610 - Simple
- 631,239 - LTR
- 477,121 - DNA
- 102,103 - Low_complexity
- 33,663 - Satellite
- 15,886 - RNA
- 6,550 - Other

## Detail class profiles

- 1,698,129 - SINE
- 1,423,257 - LINE
- 668,610 - Simple_repeat
- 624,018 - LTR
- 473,103 - DNA
- 102,103 - Low_complexity
- 33,663 - Satellite
- 7,221 - LTR?
- 6,317 - snRNA
- 4,347 - Unknown
- 4,018 - DNA?
- 3,111 - srpRNA
- 2,441 - rRNA
- 2,347 - scRNA
- 1,690 - RC
- 1,670 - tRNA
- 359 - RC?
- 154 - Unspecified

## Credits

Thanks to Arian Smit, Robert Hubley and GIRI for providing the tools and repeat libraries used to generate this track.

## References

Smit AFA, Hubley R, Green P. *RepeatMasker Open-3.0*. http://www.repeatmasker.org. 1996-2010.

Repbase Update is described in:

Jurka J. Repbase Update: a database and an electronic journal of repetitive elements. *Trends Genet*. 2000 Sep;16(9):418-420. PMID: 10973072

For a discussion of repeats in mammalian genomes, see:

Smit AF. Interspersed repeats and other mementos of transposable elements in mammalian genomes. *Curr Opin Genet Dev*. 1999 Dec;9(6):657-63. PMID: 10607616

Smit AF. The origin of interspersed repeats in the human genome. *Curr Opin Genet Dev*. 1996 Dec;6(6):743-8. PMID: 8994846
