# Supplementary material for: Mapping Retrotransposon LINE-1 Sequences into Two Cebidae Species and Homo sapiens Genomes and a Short Review on Primates
Source: Genes (Basel). 2022 Sep 27;13(10):1742. doi: 10.3390/genes13101742 (PMC9601419; doi:10.3390/genes13101742)
Supplement: Supplementary file 1 [file genes-13-01742-s001.zip › Supplementary File S2 human - RepeatMasker Repetitive Elements.pdf]

Schema for RepeatMasker - RepeatMasker Repetitive Elements

Database: hub_2004795_GCF_000001405.39 **Primary Table:** hub_2004795_repeatMasker **Data last updated:** 2022-05-14
Big Bed File: https://hgdownload.soe.ucsc.edu/hubs/GCF/000/001/405/GCF_000001405.39/bbi/GCF_000001405.39_GRCh38.p13.rmsk.bb
Item Count: 4,751,049
Format description: Repetitive Element Annotation

field	example
chrom	NW_011332701v1_alt
chromStart	3330017
chromEnd	3336395
name	L1ME3C#LINE/L1
score	252
strand	-
thickStart	3330203
thickEnd	3330777
reserved	0
blockCount	5
blockSizes	186,34,0,254,5618
blockStarts	-1,186,-1,506,-1
id	197534
description	662 25.2 10.3 1.0 NW_011332701v1_alt 3330204 3330237 (1668725) C L1ME3C LINE/L1 (186) 5937 5900 197534,662 25.2 10.3 1.0 NW_011332701v1_alt 3330524 3330777 (1668185) C L1ME3C LINE/L1

Sample Rows

chrom	chromStart	chromEnd	name	score	strand	thickStart	thickEnd	reserved	blockCount	blockSizes	blockStarts
NW_011332701v1_alt	3330017	3336395	L1ME3C#LINE/L1	252	-	3330203	3330777	0	5	186,34,0,254,5618	-1,186,-1,506,-1
NW_011332701v1_alt	3332459	3332920	EUTREP2#DNA?/hAT?	286	+	3332480	3332814	0	3	20,334,106	-1,21,-1
NW_011332701v1_alt	3333000	3333206	MIR3#SINE/MIR	341	-	3333011	3333100	0	3	11,89,106	-1,11,-1
NW_011332701v1_alt	3333684	3340275	L1MC4a#LINE/L1	304	+	3337279	3340011	0	15	3594,147,0,189,32,220,0,327,1203,250,0,27,0,19,264	-1,3595,-1,3975,-1,4311,-1,4845,-1,
NW_011332701v1_alt	3334429	3337849	L2#LINE/L2	314	+	3336438	3336526	0	3	2008,88,1323	-1,2009,-1
NW_011332701v1_alt	3335383	3338902	L2a#LINE/L2	266	-	3335391	3336429	0	5	8,236,16,564,2473	-1,8,-1,482,-1
NW_011332701v1_alt	3335559	3335870	AluY#SINE/Alu	49	+	3335640	3335863	0	3	80,223,7	-1,81,-1
NW_011332701v1_alt	3337425	3337670	AluSz#SINE/Alu	120	+	3337426	3337659	0	3	0,233,11	-1,1,-1
NW_011332701v1_alt	3337872	3337945	A-rich#Low_complexity	245	+	3337873	3337945	0	3	0,72,0	-1,1,-1
NW_011332701v1_alt	3338214	3338531	AluSp#SINE/Alu	67	+	3338215	3338529	0	3	0,314,2	-1,1,-1

RepeatMasker (hub_2004795_repeatMasker) Track Description

Description

This track shows the Repeat Masker annotations on the 28 Feb 2019 *Homo sapiens*/GCF_000001405.39_GRCh38.p13 genome assembly.

This track was created by using Arian Smit's RepeatMasker program, which screens DNA sequences for interspersed repeats and low complexity DNA sequences. The program outputs a detailed annotation of the repeats (generally available on the Downloads page). RepeatMasker uses the Repbase Update library of repeats from the Genetic Information Research Institute (GIRI). Repbase Update is described in Jurka (2000) in the Refere

Display Conventions and Configuration

Context Sensitive Zooming

This track employs a technique which chooses the appropriate visual representation for the data based on the zoom scale, and or the number of annotations currently in view. The track will automatically switch from the mo

view ('Dense' mode) if more than 500 annotations are present in the current view.

Dense Mode Visualization

In dense display mode, a single line is displayed denoting the coverage of repeats using a series of colored boxes. The boxes are colored based on the classification of the repeat (see below for legend).

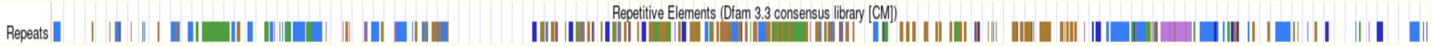

Pack Mode Visualization

In pack mode, repeats are represented as sets of joined features. These are color coded as above based on the class of the repeat, and the further details such as orientation (denoted by chevrons) and a family label are p

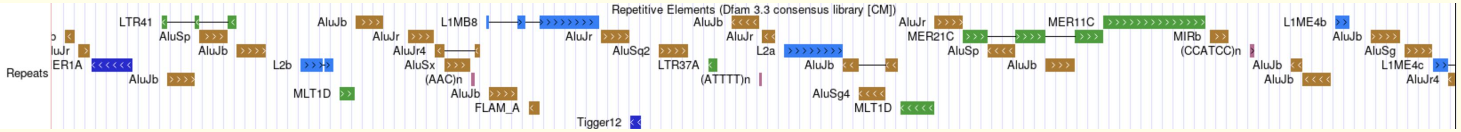

The pack display mode may also be configured to resemble the original UCSC repeat track. In this visualization repeat features are grouped by classes (see below), and displayed on seperate track lines. The repeat range
The higher the combined number of these, the lighter the shading.

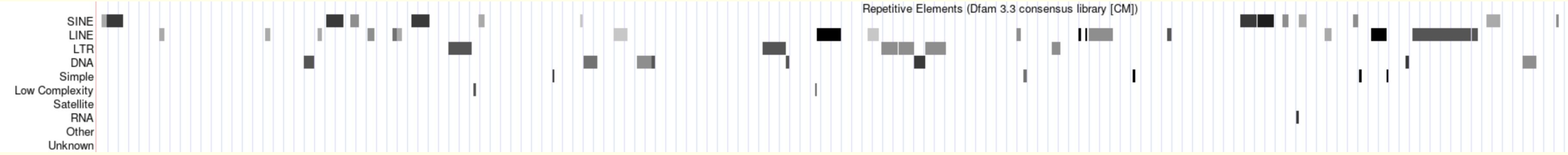

Full Mode Visualization

In the most detailed visualization repeats are displayed as chevron boxes, indicating the size and orientation of the repeat. The interior grayscale shading represents the divergence of the repeat (see above) while the outli
for where a repeat fragment originates in its consensus or pHMM model. If the length of the unaligned sequence is large, an iterruption line and bp size is indicated instead of drawing the extension to scale.

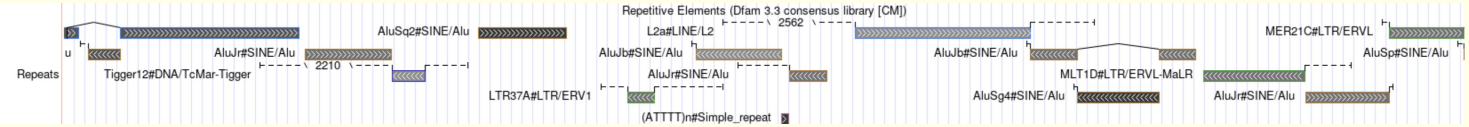

For example, the following repeat is a SINE element in the forward orientation with average divergence. Only the 5' proximal fragment of the consensus sequence is aligned to the genome. The 3' unaligned length (384bp)

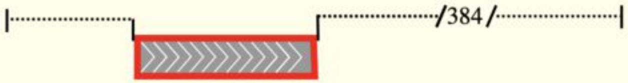

Repeats that have been fragmented by insertions or large internal deletions are now represented by join lines. In the example below, a LINE element is found as two fragments. The solid connection lines indicate that there
following the last fragment.

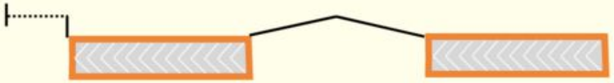

In cases where there is unaligned consensus sequence between the fragments, the repeat will look like the following. The dotted line indicates the length of the unaligned sequence between the two fragments. In this case

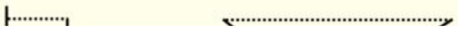

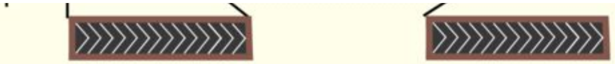

If there is consensus overlap between the two fragments, the joining lines will be drawn to indicate how much of the left fragment is repeated in the right fragment.

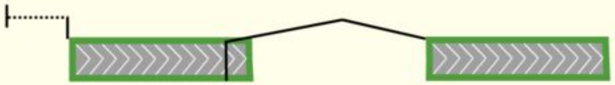

The following table lists the repeat class colors:

Color	Repeat Class
	**SINE - Short Interspersed Nuclear Element**
	**LINE - Long Interspersed Nuclear Element**
	**LTR - Long Terminal Repeat**
	**DNA - DNA Transposon**
	**Simple - Single Nucleotide Stretches and Tandem Repeats**
	**Low_complexity - Low Complexity DNA**
	**Satellite - Satellite Repeats**
	**RNA - RNA Repeats (including RNA, tRNA, rRNA, snRNA, scRNA, srpRNA)**
	**Other - Other Repeats (including class RC - Rolling Circle)**
	**Unknown - Unknown Classification**

A "?" at the end of the "Family" or "Class" (for example, DNA?) signifies that the curator was unsure of the classification. At some point in the future, either the "?" will be removed or the classification will be changed.

Methods

The RepeatMasker (www.repeatmasker.org) tool was used to generate the datasets found on this track hub.

Class profiles

- 1,891,263 - SINE
- 1,606,379 - LINE
- 779,205 - LTR
- 717,567 - Simple
- 543,873 - DNA
- 105,991 - Low_complexity
- 17,896 - Satellite
- 13,220 - Other
- 12,741 - RNA

Detail class profiles

- 1,891,263 - SINE
- 1,606,379 - LINE
- 770,871 - LTR
- 717,567 - Simple_repeat
- 539,623 - DNA

- 105,991 - Low_complexity
- 17,896 - Satellite
- 8,334 - LTR?
- 5,915 - Retroposon
- 5,352 - snRNA
- 4,868 - Unknown
- 4,250 - DNA?
- 2,043 - tRNA
- 1,935 - rRNA
- 1,926 - scRNA
- 1,859 - RC
- 1,485 - srpRNA
- 417 - RC?
- 161 - Unspecified

Credits

Thanks to Arian Smit, Robert Hubley and GIRI for providing the tools and repeat libraries used to generate this track.

References

Smit AFA, Hubley R, Green P. *RepeatMasker Open-3.0*. http://www.repeatmasker.org. 1996-2010.

Repbase Update is described in:

Jurka J. Repbase Update: a database and an electronic journal of repetitive elements. *Trends Genet*. 2000 Sep;16(9):418-420. PMID: 10973072

For a discussion of repeats in mammalian genomes, see:

Smit AF. Interspersed repeats and other mementos of transposable elements in mammalian genomes. *Curr Opin Genet Dev*. 1999 Dec;9(6):657-63. PMID: 10607616

Smit AF. The origin of interspersed repeats in the human genome. *Curr Opin Genet Dev*. 1996 Dec;6(6):743-8. PMID: 8994846
