# Supplementary material for: Mapping Retrotransposon LINE-1 Sequences into Two Cebidae Species and Homo sapiens Genomes and a Short Review on Primates
Source: Genes (Basel). 2022 Sep 27;13(10):1742. doi: 10.3390/genes13101742 (PMC9601419; doi:10.3390/genes13101742)
Supplement: Supplementary file 1 [file genes-13-01742-s001.zip › Supplementary File S3 LINE-1 sequence RM2sequpload_1661073286.align1.pdf]

```
69 8.60 1.74 1.74 NW_022436941.1 1361 1475 (2515) (AC)n#Simple_repeat 1 115 (0) c_b1s251i0 1

  NW_022436941.       1361 ACACACCCACACCCAC-CGACACACACCCACACACACA-AAATCCCACAC 1408
                                 v     v   - -        v          - v - v
  (AC)n#Simple_          1 ACACACACACACACACAC-ACACACACACACACACACACACA-CACACAC 48

  NW_022436941.       1409 CCACACACACCCACACAGATACACACACACACACACACACACACACACAC 1458
                           v         v      v i
  (AC)n#Simple_         49 ACACACACACACACACACACACACACACACACACACACACACACACACAC 98

  NW_022436941.       1459 ACACACACACACACACA 1475

  (AC)n#Simple_         99 ACACACACACACACACA 115

Matrix = Unknown
Transitions / transversions = 0.12 (1/8)
Gap_init rate = 0.04 (4 / 114), avg. gap size = 1.00 (4 / 4)


410 20.23 0.00 1.12 NW_022436941.1 1684 1773 (2217) C 7SLRNA#SINE/Alu (222) 98 10 m_b1s502i0 2

  NW_022436941.       1684 CATAGCTCACTGCAGCCTCAAACTCCTAGGCTCAAGCAATTCTCCCACCT 1733
                                 v    i     ivi       i         i  i     i
C 7SLRNA#SINE/A         98 CATAGCGCACTACAGCCCAGAACTCCTGGGCTCAAGCGATCCTCCCGCCT 49

  NW_022436941.       1734 TCGCCTCCAGTGTTGACGGGGACTACAGGCGCAAGCCCCC 1773
                           iv      v v  v - v              iv   v
C 7SLRNA#SINE/A         48 CAGCCTCCCGAGTAG-CTGGGACTACAGGCGCGCGCCACC 10

Matrix = 20p37g.matrix
Kimura (with divCpGMod) = 19.55
Transitions / transversions = 1.00 (9/9)
Gap_init rate = 0.01 (1 / 89), avg. gap size = 1.00 (1 / 1)


14 11.65 0.00 3.45 NW_022436941.1 1783 1812 (2178) (TTTAC)n#Simple_repeat 1 29 (0) m_b1s252i0 3

  NW_022436941.       1783 TTTACTTTCCTTTACTTTTATTTTATTTTA 1812
                                   v      -    i    i
  (TTTAC)n#Simp          1 TTTACTTTACTTTAC-TTTACTTTACTTTA 29

Matrix = Unknown
Transitions / transversions = 2.00 (2/1)
Gap_init rate = 0.03 (1 / 29), avg. gap size = 1.00 (1 / 1)


468 21.35 0.78 9.24 NW_022436941.1 1813 1941 (2049) C PB1D7#SINE/Alu (0) 120 2 m_b1s502i1 4
```

```
  NW_022436941.       1813 TTTTACTTTCTTGTACAGAAAGGGATCTCACCATGTTGACCAGGCTGATC 1862
                               ii   i   ---   v    -      iv   v v        ii
C PB1D7#SINE/Al        120 TTTTGTTTTTTTG---AGACAGGG-TCTCACTCTGTAGCCCAGGCTGGCC 75

  NW_022436941.       1863 TGGAACCTGGGCTCA-GGCTAGCCTCCTTCTTCAGCCTCCCAAAGTGCTA 1911
                            v    ------   -   v v      v i          i -     i
C PB1D7#SINE/Al         74 TCGAAC------TCACGGCGATCCTCCTGCCTCAGCCTCCCGA-GTGCTG 32

  NW_022436941.       1912 GGATTACAAGTGTGAACCACAGTGCTCAGC 1941
                                   i i    i    vii  i i
C PB1D7#SINE/Al         31 GGATTACAGGCGTGAGCCACCACGCCCGGC 2

Matrix = 20p37g.matrix
Kimura (with divCpGMod) = 21.01
Transitions / transversions = 1.78 (16/9)
Gap_init rate = 0.09 (12 / 128), avg. gap size = 1.00 (12 / 12)

483 25.00 1.77 0.88 NW_022436941.1 1829 1941 (2049) C PB1#SINE/Alu (12) 115 2 m_b1s502i2 4

  NW_022436941.       1829 AGAAAGGGATCTCACCATGTTGACCAGGCTGATCTGGAAC--CTGGGCTC 1876
                              v    -    i i      v        ii   v    --    i
C PB1#SINE/Alu         115 AGACAGGG-TCTCGCTATGTTGCCCAGGCTGGCCTCGAACTCCTGGACTC 67

  NW_022436941.       1877 AGGCTAGCCTCCTTCTTCAGCCTCCCAAAGTGCTAGGATTACAAGTGTGA 1926
                            i iv v      v i          i ivv   i   i    i i   v
C PB1#SINE/Alu          66 AAGTGATCCTCCTGCCTCAGCCTCCCGAGTAGCTGGGACTACAGGCGTGC 17

  NW_022436941.       1927 ACCACAGTGCTCAGC 1941
                           i    v i  i i
C PB1#SINE/Alu          16 GCCACCGCGCCCGGC 2

Matrix = 20p37g.matrix
Kimura (with divCpGMod) = 23.78
Transitions / transversions = 1.80 (18/10)
Gap_init rate = 0.02 (2 / 112), avg. gap size = 1.50 (3 / 2)


27 18.49 5.19 2.53 NW_022436941.1 2075 2151 (1839) (TA)n#Simple_repeat 1 79 (0) m_b1s252i1 5

  NW_022436941.       2075 TATTTATA-ATGATTTATATATCATGTACATATATATATTTTTATGTACA 2123
                              v    -  -  v       -  i  i          v v   i  i
  (TA)n#Simple_          1 TATATATATAT-ATATATATAT-ATATATATATATATATATATATATATATA 48

  NW_022436941.       2124 TATAT-TTTATATA-ATAGATACA-ATTTAT 2151
                                - v      -   v   i -  v
  (TA)n#Simple_         49 TATATATATATATATATATATATATATATAT 79
```

```
Matrix = Unknown
Transitions / transversions = 0.71 (5/7)
Gap_init rate = 0.08 (6 / 76), avg. gap size = 1.00 (6 / 6)

19 24.83 5.62 2.17 NW_022436941.1 2076 2164 (1826) (ATATATA)n#Simple_repeat 1 92 (0) m_b1s252i2 5

  NW_022436941.       2076 ATTTATAATGAT-TTATATATCATGTA-CATATAT-ATAT-TTTTATGTA 2121
                             v      -  - v      v  i  -v      -    - vv   i
  (ATATATA)n#Si          1 ATATATAAT-ATATAATATATAATATATAATATATAATATATAATATATA 49

  NW_022436941.       2122 CATATATTTTATATAATAGATACAATTTATTA-ACAATTTACAA 2164
                           -      vv         v   vv  v  v  - i   v  i
  (ATATATA)n#Si         50 -ATATATAATATATAATATATAATATATAATATATAATATATAA 92

Matrix = Unknown
Transitions / transversions = 0.29 (4/14)
Gap_init rate = 0.08 (7 / 88), avg. gap size = 1.00 (7 / 7)


369 30.36 4.32 2.42 NW_022436941.1 3422 3583 (407) C L4_C_Mam#LINE/RTE-X (515) 4623 4459 m_b1s551i0 6

  NW_022436941.       3422 GGGAAGCTAAACTAACATGGTCTAATTATTCTAACCTCTGTTCACCTAAC 3471
                              i      i?   ii     i iv   vi?ivii    v v i
C L4_C_Mam#LINE       4623 GGGGAGCTAAGNTAATGTGGTCCAGGTATGTNGCTTTCTGGTGATCTAAC 4574

  NW_022436941.       3472 TTGATTTAGGAACAAATCATTGAGAGT-TAGAGCA-TGAATAGTCAAGAT 3519
                                vi   i i i v v v    i -     v -  i  i  i iv
C L4_C_Mam#LINE       4573 TTGATACAGGGATAGAGCTTAGAGAATCTAGAGGAATGGATGGTTAGCAT 4524

  NW_022436941.       3520 ---CTTTAGACCATATCTC--AAATATAAGTGATCTGAATCAACTCTGTT 3564
                           --- i      i  v    --      v   vi   v ii i v-  v
C L4_C_Mam#LINE       4523 GTCCCTTAGACTATCTCTCTCAAATATCAGTTGTCTCAGCCGAG-CTTTT 4475

  NW_022436941.       3565 TCCAGTTTGAGCTGAATAG 3583
                           vv   ---      i
C L4_C_Mam#LINE       4474 GGCAG---GAGCTGGATAG 4459

Matrix = 20p37g.matrix
Kimura (with divCpGMod) = 37.46
Transitions / transversions = 1.42 (27/19)
Gap_init rate = 0.05 (8 / 161), avg. gap size = 1.38 (11 / 8)
```
