# Supplementary figures and images for: Mapping Retrotransposon LINE-1 Sequences into Two Cebidae Species and Homo sapiens Genomes and a Short Review on Primates
Source: Genes (Basel). 2022 Sep 27;13(10):1742. doi: 10.3390/genes13101742 (PMC9601419; doi:10.3390/genes13101742)

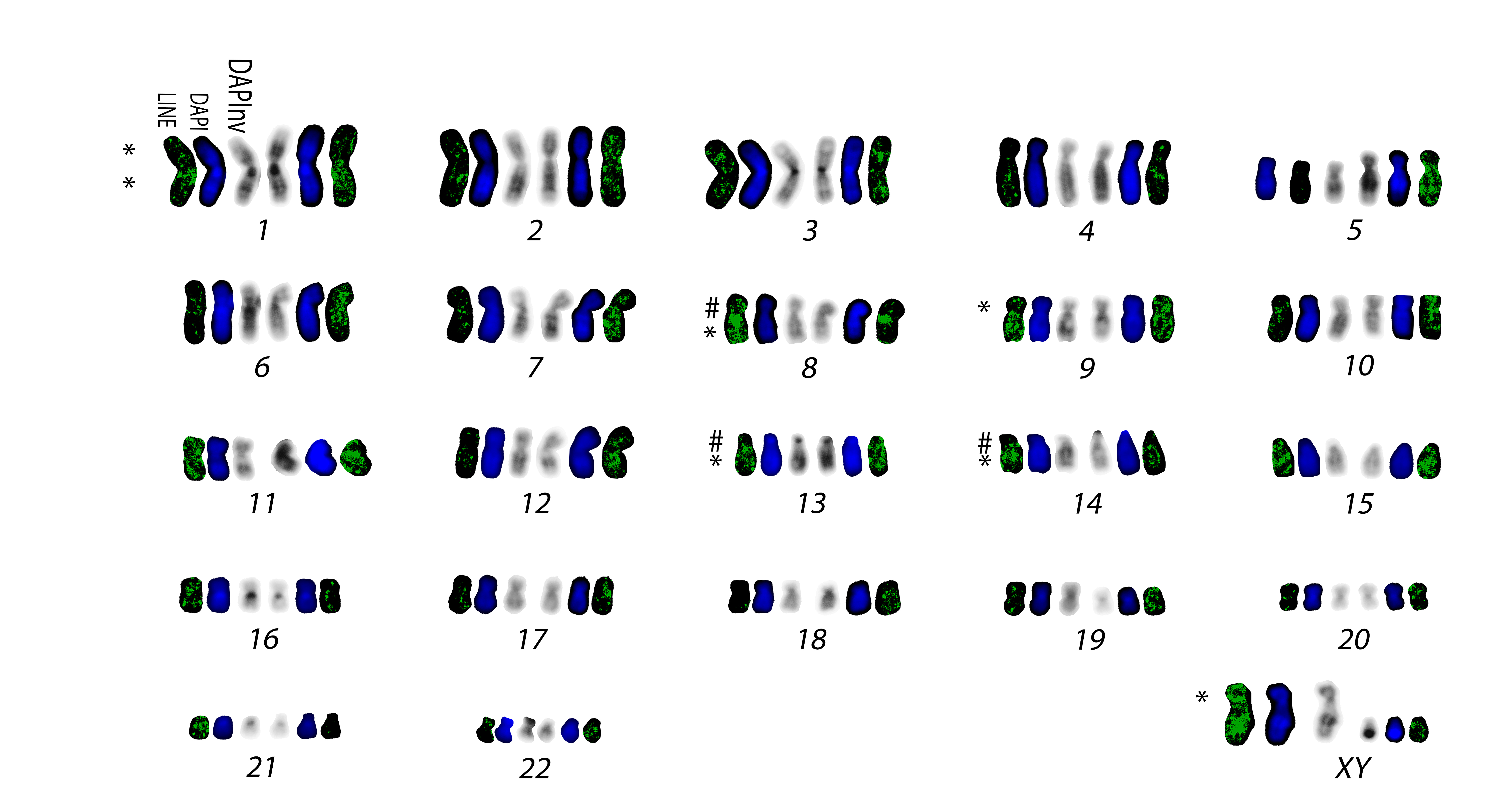

Supplement: Supplementary file 1 [file genes-13-01742-s001.zip › Supplementary Figure S1 Ricostruzione HSA 5519.jpg]
